# Innovations in Healthy Aging Around the Globe

**DOI:** 10.1093/geroni/igaf122.843

**Published:** 2025-12-31

**Authors:** Chien-Ching Li, Tung Sung Tseng, Darren Liu

## Abstract

As populations continue to age, the demand for effective healthcare and social support strategies becomes increasingly urgent. This symposium presents five studies that highlight significant advancements in aging-related care and emphasize the importance of tailored interventions to address the needs of older adults in the United States, Taiwan, and Nigeria. The first study examines the impact of social function, fatigue, and smoking status on the well-being of American prostate cancer survivors, offering insights to improve patient care. The second study introduces an innovative decision-support tool designed to assist Chinese American family caregivers in navigating dementia care options effectively. The third study explores the shift to virtual healthcare during the COVID-19 pandemic and its impact on provider satisfaction, underscoring the need for enhanced virtual communication training to ensure high-quality care for older adults in the U.S. The fourth study identifies key barriers to diabetes management among older adults in Nigeria, highlighting the need for improved healthcare access and support systems. The fifth study investigates Dance Movement Therapy as a non-pharmacological intervention that enhances bodily awareness, emotional expression, and social connection, fostering engagement, resilience, and a holistic, person-centered approach to care. Overall, this symposium presents cutting-edge research addressing the complex challenges faced by older adults. It offers valuable perspectives on aging-related healthcare and social support, fostering discussions on innovative, culturally responsive interventions for diverse aging populations. International Comparisons of Healthy Aging Interest Group Sponsored Symposium

